# Maternal rheumatoid arthritis and risk of autism in the offspring

**DOI:** 10.1017/S0033291723000855

**Published:** 2023-11

**Authors:** Weiyao Yin, Mattias Norrbäck, Stephen Z. Levine, Natalia Rivera, Joseph D. Buxbaum, Hailin Zhu, Benjamin Yip, Abraham Reichenberg, Johan Askling, Sven Sandin

**Affiliations:** 1 Department of Medical Epidemiology and Biostatistics, Karolinska Institutet, Stockholm, Sweden; 2 Department of Obstetrics and Gynecology, West China Second University Hospital, Sichuan University, Chengdu, China; 3 Clinical Epidemiology Division, Department of Medicine, Karolinska Institutet, Stockholm, Sweden; 4 School of Public Health, University of Haifa, Haifa, Israel; 5 Department of Medicine Solna, Respiratory Medicine Division, Karolinska Institutet, Stockholm, Sweden; 6 Rheumatology Division, Department of Medicine Solna, Karolinska Institutet, Karolinska University Hospital, Stockholm, Sweden; 7 Seaver Autism Center for Research and Treatment at Mount Sinai, Icahn School of Medicine at Mount Sinai, New York, USA; 8 Jockey Club School of Public Health and Primary Care, the Chinese University of Hong Kong, Hong Kong SAR, China; 9 Department of Psychiatry, Icahn School of Medicine at Mount Sinai, New York, USA; 10 Rheumatology, Theme Inflammation and Ageing, Karolinska University Hospital, Stockholm, Sweden

**Keywords:** Autism, autoimmune, cohort, epidemiology, joint pain, prenatal risk, rheumatoid arthritis, seronegative

## Abstract

**Background:**

Maternal Rheumatoid Arthritis (RA) is suggested to increase the risk of Autism Spectrum Disorder (ASD) in the offspring, mainly through inflammation/autoimmunity, but the association is unclear. A prospective population-based cohort study was implemented to examine the association between maternal RA and offspring ASD.

**Methods:**

We included all children born alive in Sweden from 1995 to 2015, followed up through 2017. Diagnoses of ASD and RA were clinically ascertained from National Patient Register. We quantified the association by hazard ratios (HR) and two-sided 95% confidence intervals (CI), from Cox regression after detailed adjustment for potential confounders. We examined RA serostatus, etiological subgroups and the timing of exposure. To closer examine the underlying mechanism for the association, we included a *negative* control group for RA, arthralgia, with similar symptomology as RA but free from inflammation/autoimmunity.

**Results:**

Of 3629 children born to mothers with RA, 70 (1.94%) were diagnosed with ASD, compared to 28 892 (1.92%) of 1 503 908 children born to mothers without RA. Maternal RA before delivery was associated with an increased risk of offspring ASD (HR = 1.43, 95% CI 1.11–1.84), especially for seronegative RA (HR = 1.61, 95% CI 1.12–2.30). No similar association was observed for paternal RA, maternal sisters with RA, or RA diagnosed after delivery. Maternal arthralgia displayed as high risks for offspring ASD as did maternal RA (HR = 1.41, 95% CI 1.24–1.60).

**Conclusions:**

In Sweden, maternal RA before delivery was associated with an increased risk of offspring ASD. The comparable association between maternal arthralgia and ASD risk suggests other pathways of risk than autoimmunity/inflammation, acting jointly or independently of RA.

## Introduction

Rheumatoid arthritis (RA) is a common chronic inflammatory condition, with a prevalence of 0.5–1.0% among women of childbearing age (Garrick, 2017). Maternal RA before delivery has been associated with increased risks of perinatal complications (Bermas & Sammaritano, 2015; Ince-Askan & Dolhain, 2015; Langen, Chakravarty, Liaquat, El-Sayed, & Druzin, 2014) and later neurological and neurodevelopmental disorders in the offspring (Dalsgaard, Waltoft, Leckman, & Mortensen, 2015; Instanes et al., 2017; Mataix-Cols et al., 2018; Nielsen et al., 2021).

Autism Spectrum Disorder (ASD), is a chronic neurodevelopmental disorder that affects 1–2% of children worldwide (Baxter et al., 2015; Buxbaum & Hof, 2013; Elsabbagh et al., 2012). Genetic factors have a substantial role in the etiology of ASD (Bai et al., 2019; Sandin et al., 2014), but non-genetic factors are also important (Yip et al., 2018). While inflammatory and immune-related factors, such as inflammatory perturbations in utero and immune activity during pregnancy, have been implicated in the non-genetic etiology of ASD (Han, Patel, Jones, & Dale, 2021; Voineagu et al., 2011), previous studies on the associations between maternal RA during pregnancy and ASD in the offspring are few, and have reported mixed results (Atladóttir et al., 2009; Comi, Zimmerman, Frye, Law, & Peeden, 1999; Croen et al., 2019; Rom et al., 2018; Tsai et al., 2018). Methodological limitations such as lack of ability to separate maternal RA before and after birth of the child (Rom et al., 2018), small sample size (Croen et al., 2019; Tsai et al., 2018), and lack of consideration of important potential confounding factors further limit generalizability. In this study, we aim to test the hypothesis that maternal RA before delivery is associated with an increased risk of ASD in the offspring.

To overcome the methodological limitations of previous research we utilized a population-based sample of 1.5 million births. We studied the timing of maternal RA (separately before and after delivery), made adequate control of confounding factors, and examined potential effect size moderating factors, including preterm birth (Persson et al., 2020; Rom et al., 2014), smoking (Hedström, Stawiarz, Klareskog, & Alfredsson, 2018; Rosen, Lee, Lee, Yang, & Burstyn, 2015), and obesity (Dar et al., 2018; Surén et al., 2014). RA is a heterogeneous condition, and the presence of rheumatoid factor or antibodies to citrullinated peptides (‘seropositive’) defines an entity that is distinct from the absence of these markers (‘seronegative’). The current study is the first to examine RA serostatus in relation to offspring ASD. Lastly, to examine the specificity of RA in the association with ASD, never done before, we included a large comparison group with similar symptomology as RA, i.e., arthralgia, which includes other non-inflammatory states of joint problems such as unspecific joint pain.

## Methods

### Data sources and study population

The study population comprises all children born alive in Sweden in 1995–2015, to Swedish parents, as recorded in the *Swedish Medical Birth Register* (*MBR*) (Cnattingius, Ericson, Gunnarskog, & Källén, 1990). *MBR* records maternal and child characteristics of all deliveries in Sweden since 1973, including gestational age, estimated at the early second-trimester ultrasound examination or, in less than 1% of births, using the date of last menstrual period. First-degree relatives were identified from the *Swedish Multigeneration Register* (Ekbom, 2011). The parental number of completed school years was extracted from the *Education Register.* Parental yearly income was obtained from the *Swedish Government Tax Register*. Inpatient psychiatric diagnoses since 1973, somatic diagnoses since 1987, and outpatient visits since 2001 were recorded in the *Swedish National Patient Register* (*NPR*) (Ludvigsson et al., 2011), assigned by clinical specialists according to the International Classification of Diseases (ICD). The data quality of the NPR has been verified (Byrne, Regan, & Howard, 2005), and validated for the diagnosis of ASD (Sandin et al., 2016) and RA (Waldenlind, Eriksson, Grewin, & Askling, 2014). Date for emigration and death was derived from the *Migration Register* and the *Statistics Sweden Total Population Register*, respectively. The Swedish identifying number for each citizen (Ludvigsson et al., 2011) was used to link the individual-level information from different registers.

### Clinical diagnoses: ASD, RA and arthralgia

Clinical diagnoses were ascertained from the NPR (ICD codes in online Supplementary eTable 1). In Sweden, all infants and preschool children undergo routine medical examinations and developmental assessments (motor skills, language, cognitive and social development). Children with suspected ASD are referred to a child psychiatry unit or habilitation service. We defined RA as presence of at least one RA diagnosis any time prior to the delivery. In sensitivity analyses, we used more stringent diagnostic criteria requiring at least two RA diagnoses. In the ICD system, serostatus can be derived from the RA code. We used the mother's all available RA diagnoses up to delivery, and defined seropositive or seronegative status as the most common status. If equally frequent, we used the last diagnosis to define the RA subtype.

The key postulated mechanism in this study involves fetal exposure to inflammation/autoimmunity in RA. In order to challenge this mechanism we introduced a negative control diagnosis, unspecific joint pain (arthralgia). Arthralgia in the absence of RA or other defined inflammatory origins serves as a reasonable negative control in the sense that it typically shares many of the symptoms of RA (e.g. pain) save for the inflammatory milieu and autoimmune origin. Diagnosis of RA and arthralgia are mostly mutually exclusive. The vast majority of individuals with arthralgia as their first diagnosis does not have RA later and reversed (online Supplementary eFig. 1).

### Other covariates

We included offspring year of birth, maternal and paternal age, income (Swedish Krona, SEK), education (<9 years of primary education, 9 years of primary education, 1–2 years of secondary school education, 3 years of secondary school education, 1–2 years of postgraduate education, ⩾3 years of postgraduate education, PhD education), and psychiatric disease history (yes/no) at the time of delivery. From MBR we obtained maternal body mass index (BMI; kg/m^2^) at first antenatal visit, maternal smoking during pregnancy (yes/no), offspring gestational age at birth (weeks) and offspring sex as potential confounding or modifying factors.

### Statistical methods

We quantified the association between maternal RA before delivery and offspring ASD risk by hazard ratios (HR) and associated two-sided 95% confidence intervals (CI), from Cox proportional hazards models, together with ASD incidence rate (cases per 100 000 person-years). Each child was followed from age two, as ASD cannot be reliably diagnosed earlier, until the first ASD diagnosis, emigration, death, or the 31^st^ December 2017, whichever came first.

The primary analyses consisted of fitting a sequence of models with increasing adjustment for potential confounding. Continuous variables were modeled as natural cubic splines (Benedetti & Abrahamowicz, 2004), with five degrees of freedom to allow for non-linear relations. Variables from mothers and fathers were included separately in the model. In the first, ‘crude’, model we adjusted for birth year. Next, we adjusted for maternal and paternal age (Sandin et al., 2016), education and income, at delivery. In the third model, we additionally adjusted for maternal and paternal psychiatric history at delivery (Euesden et al., 2017; Jokiranta et al., 2013). Inverse Kaplan–Meier curves (crude and adjusted) were used to depict the cumulative incidence of ASD.

The proportional hazards assumption of the Cox regression was visually examined by Schoenfeld residuals (Grambsch & Therneau, 1994). All statistical tests were performed on the two-sided 5% level of significance. We did not adjust the *p* values for the multiplicity of statistical tests. However, the primary hypothesis is composed of a single test (mothers with RA *v.* mothers without RA). Robust standard errors adjust for potential correlations between siblings (Lin & Wei,, 1989).

### Sensitivity analyses

We repeated the primary analyses requiring at least two recorded RA diagnoses. We examined narrowly defined autistic disorders (AD; online Supplementary eTable 1). We restricted analysis to singletons and children without malformations separately. To address potential concerns about sparse data and small sample biases of the parameter estimates, we applied Firth's penalized likelihood (Firth, 1993; Heinze & Schemper, 2001).

### Supplementary analyses

(1) To examine the specificity of maternal RA and potential influences of familial factors, we examined ASD risk in offspring of fathers with RA, compared to fathers free from RA, before the time of their spouse's delivery. (2) To examine the influences of inheritance by RA, among mothers free from RA diagnosis, we compared the ASD risk in the offspring of mother's sisters with RA to the ASD risk in the offspring of mother's sisters without RA. If ASD risk is inherited in families with RA, the offspring of the full sisters with RA would be expected with increased risk as well. (3) To examine ASD risk in relation to the timing of RA, we calculated incidence rate ratios (IRR) with maternal RA as a time-varying exposure in Poisson regression model. Here, we compared the ASD incidence in offspring of mothers with RA first diagnosed before delivery, in mothers with RA first diagnosed after delivery, and in mothers without RA. (4) To assess if the association between maternal RA and ASD is specific to RA or present also in a negative control group, i.e. maternal arthralgia, we fitted a Poisson regression model with incident maternal RA and maternal arthralgia present before delivery as well as time-varying after delivery. Furthermore, we compared the risk for maternal sisters with arthralgia, to mothers without arthralgia or RA. (5) We compared ASD risk by maternal seronegative RA and seropositive RA. (6) We estimated risk separately by offspring sex. (7) Preterm birth, as a risk factor for ASD (Persson et al., 2020), is more common in mothers with RA (Rom et al., 2014). We, therefore, examined the modifying role of gestational age. First, we repeated analyses in subgroups of children born term (37–40 weeks), preterm (<37 weeks), and post-term (⩾41 weeks). Next, we performed a mediation analysis of preterm *v.* term-born, and post-term *v.* term born by approximating our Cox models with logistic regression, and fitting Natural Effects Models (VanderWeele, 2014; VanderWeele, 2015) (online Supplementary eTable 9). (8) Smoking has been suggested as a risk factor for maternal RA (Hedström et al., 2018), and sometimes for offspring ASD (Rosen et al., 2015). We therefore estimated the effect modification of maternal smoking by introducing an interaction term between maternal smoking and RA. (9) Obesity is a risk factor for maternal RA (Dar et al., 2018), and potentially for offspring ASD (Surén et al., 2014).We therefore included an interaction term between maternal BMI and RA, and assessed strata-specific ASD risk to underweight (BMI <18.5 kg/m^2^), normal weight (BMI = 18.5–24.9 kg/m^2^), overweight (BMI = 25–29.9 kg/m^2^) and obese (BMI > 30 kg/m^2^) mothers.

Statistical analyses were performed using SAS software version 9.4 (SAS institute Inc, Cary, NC, USA), using proc phreg and proc glimmix.

## Results

Of the 1 521 184 children, we excluded 7245 (0.34%) who died, emigrated or had an ASD diagnosis before age two. Only 6402 (0.42%) had missing data on primary covariates and were excluded from analysis. Thus, our analytic cohort comprised 1 507 537 children, contributing 15 408 744 person-years of follow-up (online Supplementary eFig. 2). 3629 (0.24%) of mothers were diagnosed with RA at least once before delivery, and 2468 (68%) two times or more. During an average 10-year follow-up, 70 (1.94%) of children born to mothers diagnosed with RA before delivery were later diagnosed with ASD, compared to 28 892 (1.92%) to mothers free from RA before delivery. Children of mothers with RA before delivery tended to be born in recent years and were more likely to be preterm, and their parents were older, better educated, had a higher income, and had a higher prevalence of psychiatric disease before delivery (Table 1, online Supplementary eFig. 3).

**Table 1.** Cohort description

Characteristics	Mothers with RA	Mothers without RA
	Number of children (%)	Number of children (%)
RA serostatus	3629 (0.24%)	1 503 908 (99.76%)
Seronegative	1741 (47.97%)	–
Seropositive	1888 (52.03%)	–
Offspring sex (male, %)	1813 (49.96%)	773 899 (51.46%)
Follow-up years (median, Q1–Q3)	6.3 (3.1-10.3)	9.9 (5.0-15.3)
Birth year	–	–
1995–1999	222 (6.12%)	341 747 (22.72%)
2000–2004	557 (15.35%)	349 013 (23.21%)
2005–2009	1115 (30.72%)	370 222 (24.62%)
2010–2015	1735 (47.81%)	442 926 (29.45%)
Gestational week	–	–
<37 week	370 (10.20%)	87 982 (5.85%)
37–40 week	2572 (70.87%)	1 035 528 (68.86%)
>40 weeks	687 (18.93%)	380 398 (25.29%)
Maternal age before delivery	–	–
<20	13 (0.36%)	12 620 (0.84%)
20-29	934 (25.74%)	600 850 (39.95%)
30-39	2412 (66.46%)	830 173 (55.20%)
40-49	269 (7.41%)	60 183 (4.00%)
⩾ 50	1 (0.03%)	82 (0.01%)
Paternal age before delivery	–	–
<20	3 (0.08%)	4098 (0.27%)
20–29	628 (17.31%)	404 051 (26.87%)
30–39	2384 (65.69%)	909 715 (60.49%)
40-49	572 (15.76%)	173 064 (11.51%)
⩾50	42 (1.16%)	12 980 (0.86%)
Maternal psychiatric history	495 (13.64%)	122 270 (8.13%)
Paternal psychiatric history	259 (7.14%)	79 623 (5.29%)
Maternal yearly income (median, Q1–Q3)	159 531 (121 616–211 382)	136 531 (103 746–182 841)
Paternal yearly income (median, Q1–Q3)	253 322 (189 853–327 708)	212 725 (153 652–286 075)
Maternal education attainment	–	–
<9 years in primary school	6 (0.17%)	2096 (0.14%)
9 years in primary school	267 (7.36%)	123 020 (8.18%)
1–2 years in secondary school	482 (13.28%)	260 931 (17.35%)
3 years in secondary school	1002 (27.61%)	428 818 (28.51%)
1–2 years of postgraduate education	460 (12.68%)	215 132 (14.30%)
⩾ 3 years of postgraduate education	1374 (37.86%)	464 103 (30.86%)
PhD	38 (1.05%)	9808 (0.65%)
Paternal education attainment	–	–
<9 years in primary school	6 (0.17%)	5185 (0.34%)
9 years in primary school	291 (8.02%)	153 864 (10.23%)
1–2 years in secondary school	761 (20.97%)	380 939 (25.33%)
3 years in secondary school	1074 (29.59%)	410 798 (27.32%)
1–2 years of postgraduate education	588 (16.20%)	220 046 (14.63%)
⩾ 3 years of postgraduate education	853 (23.51%)	316 456 (21.04%)
PhD	56 (1.54%)	16 620 (1.11%)

Abbreviations. Q1, 1st quartile (25th percentile); Q3, 3rd quartile (75th percentile); RA, Rheumatoid Arthritis.

### Maternal RA before delivery and offspring ASD

**Figure 1.**
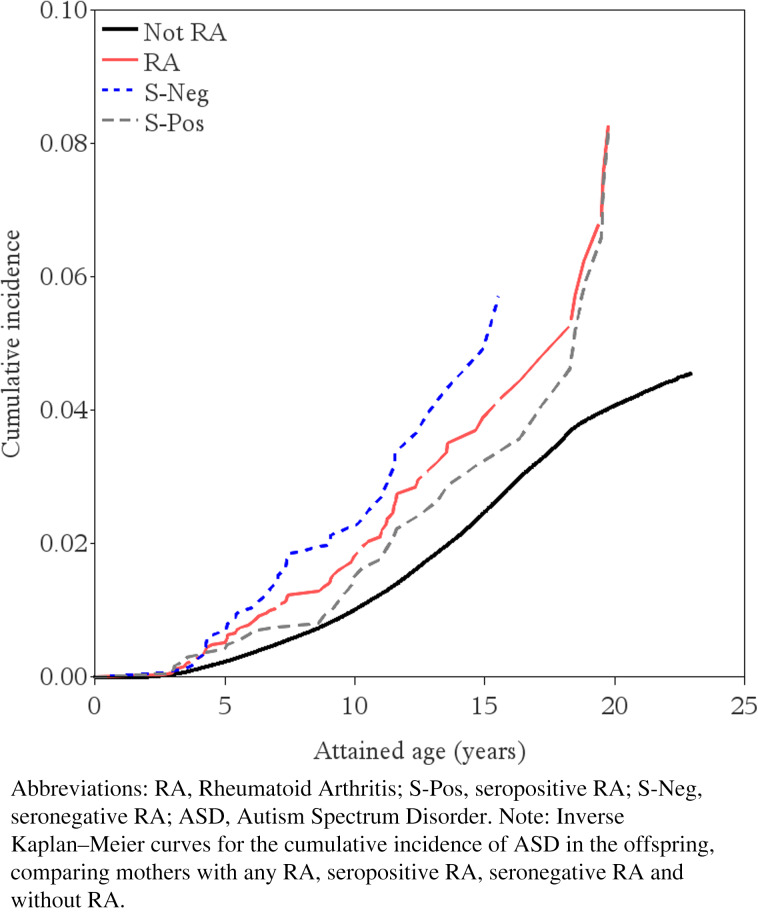
Inverse Kaplan–Meier curves for mothers with and without RA before delivery, and by seronegative and seropositive RA separately.

In the crude model, the HR for the association between maternal RA before delivery and offspring ASD was 1.45 (95% CI 1.13–1.85; 273 *v.* 188 ASD per 100 000 person-years). Adjusting for maternal and paternal age, socioeconomic status, and psychiatric history, the HR was estimated at 1.43 (95% CI 1.11–1.84, Table 2; Fig. 1; online Supplementary eFig. 4). There was no evidence for non-proportional hazards (online Supplementary eFig. 5).

**Table 2.** Relative risk of ASD in offspring of mothers, fathers and full-sisters of mothers with RA before delivery

Analysis group	ASD rate (per 100 000 person year)	ASD cases (person years)	Number of subjects	Model 1 HR (95% CI)	Model 2 HR (95% CI)	Model 3 HR (95% CI)
RA diagnosis						
Maternal RA	272.9	70 (25 652)	3629	1.45 (1.13–1.85)	1.46 (1.14–1.87)	1.43 (1.11–1.84)
No maternal RA	187.8	28 892 (15 383 092)	1 503 908	Reference	Reference	Reference
Paternal RA	177.4	13 (7327)	1004	0.94 (0.55–1.62)	0.89 (0.52–1.54)	0.88 (0.51–1.51)
No paternal RA	188.0	28 949 (15 401 417)	1 506 533	Reference	Reference	Reference
Maternal sister's RA^a^	232.2	40 (17 230)	1746	1.21 (0.87–1.69)	1.21 (0.87–1.69)	1.20 (0.86–1.68)
No maternal RA	187.8	28 845 (15 361 222)	1 502 081	Reference	Reference	Reference

Abbreviations: RA, Rheumatoid Arthritis; ASD, Autism Spectrum Disorders; HR Hazard Ratio; CI 95% Confidence Interval.

Note: Relative risks were quantified by hazard ratios with their associated two-sided 95% confidence interval fitting Cox proportional hazard regression models with robust standard errors.

Model 1: Adjusted for birth year by natural cubic splines with 5 knots; Model 2: Additionally adjusted for maternal and paternal age at delivery (by natural cubic splines), maternal and paternal education at delivery (categorized; < 9 years of primary education, 9 years of primary education, 1–2 years of secondary school education, 3 years of secondary school education, 1-2 years of postgraduate education, ⩾ 3 years of postgraduate education, PhD education), maternal and paternal income at delivery (by natural cubic splines); Model 3: Additionally adjusted for maternal and paternal psychiatric diagnoses at delivery (yes/ no).

a Children of full sisters to mothers with RA before delivery *v.* children of the remaining RA-free mothers.

### Sensitivity analyses

The estimated HRs were robust to changes in the RA definition (HR = 1.44; 95% CI 1.05–1.98 for ⩾2 diagnoses); when restricted to AD (HR = 1.50; 95%CI 1.09–2.07) (online Supplementary eTable 2); when excluding twin births (online Supplementary eTable 3) or malformation (online Supplementary eTable 4); when Firth's penalized likelihood was applied (online Supplementary eTable 5).

### Supplementary analysis

Offspring of fathers with RA before delivery (HR = 0.88, 95%CI 0.51–1.51), and offspring of full sisters of mothers with RA before delivery (HR = 1.20, 95%CI 0.86–1.68) did not have increased ASD risk (Table 2). Compared to postnatal RA, the ASD risk for children was higher for prenatal RA exposure (IRR = 1.56, 95%CI 1.16–2.09, RA as a time-varying exposure, Table 3). Compared to offspring of RA-free mothers, offspring of mothers with a first RA diagnosis after delivery was not at increased risk of ASD (IRR = 1.09, 95%CI 0.92–1.30).

**Table 3.** Risk of ASD in offspring of a mother with RA, by RA serostatus and the timing of exposure

Analysis group	ASD rate (per 100 000 person year)	ASD cases (person years)	Model 1 HR (95% CI)	Model 3 HR (95% CI)
By serostatus^a^				
Seronegative	314.8	35 (11 119)	1.65 (1.16–2.37)	1.61 (1.12–2.30)
Seropositive	240.8	35 (14 534)	1.28 (0.91–1.82)	1.29 (0.91–1.82)
Non-RA	187.8	28 892 (15 383 092)	Reference	Reference
Seronegative v. seropositive	–	–	1.29 (0.78–2.12)	1.25 (0.76–2.05)
By exposure timing^b^				
RA before delivery	278.5	70 (25 133)	1.48 (1.17–1.87)	1.70 (1.35–2.15)
RA after delivery	290.4	125 (43 047)	1.17 (0.98–1.40)	1.09 (0.92–1.30)
Non-RA	187.7	28 767 (15 328 041)	Reference	Reference
RA before v. RA after delivery	–	–	1.26 (0.94–1.69)	1.56 (1.16–2.09)

Abbreviations: RA, Rheumatoid Arthritis; ASD, Autism Spectrum Disorders; HR, Hazard Ratio; CI, Confidence interval.

Note: ^a^To decide serostatus status, we used the mother's all available RA diagnoses up to delivery, and defined seropositive or seronegative status as the most common type among these. If equally frequent, we used the last diagnosis to define the RA subtype.

Note: Relative risks were quantified by hazard ratios with 95% confidence intervals fitting Cox proportional hazard regression models with robust standard errors. Model 1: Adjusted for birth year by natural cubic splines with 5 knots; Model 3: Additionally adjusted for maternal and paternal age at delivery (by natural cubic splines), maternal and paternal education at delivery (categorized; < 9 years of primary education, 9 years of primary education, 1–2 years of secondary school education, 3 years of secondary school education, 1–2 years of postgraduate education, ⩾ 3 years of postgraduate education, PhD education), maternal and paternal income at delivery (by natural cubic splines), maternal and paternal psychiatric diagnoses at delivery (yes/ no).

^b^Relative risks with 95% confidence intervals were calculated using Poisson regression models, including RA diagnosis as a time-varying exposure. The cases and person-years are summarized at the end of follow-up. Model 1: Adjusted for birth year by natural cubic splines with 5 knots; Model 3: Additionally adjusted for maternal and paternal age at delivery (by natural cubic splines), maternal and paternal education at delivery (categorized), maternal and paternal income at delivery (by natural cubic splines), maternal and paternal psychiatric diagnoses at delivery (yes/ no).

Among offspring of mothers with arthralgia before delivery, 240 (incidence rate = 281 per 100 000 person-years) were diagnosed with ASD, compared to 27 485 (incidence rate = 183 per 100 000 person-years) of mothers without arthralgia and RA. The relative risk of ASD was estimated at 1.41 (95% CI 1.24–1.60) for maternal arthralgia before delivery, and 1.36 (95% CI 1.27–1.44) for maternal arthralgia after delivery (online Supplementary eTable 6, eFig. 6). Offspring of full sisters to mothers with arthralgia before delivery was not at increased ASD risk (online Supplementary eTable 7).

Seronegative RA (HR = 1.61, 95% CI 1.12–2.30), but not seropositive RA (HR = 1.29, 95% CI 0.91–1.82), was statistically significantly associated with increased ASD risk (Table 3). The interaction between offspring sex and maternal RA was not statistically significant (*p* value = 0.22; online Supplementary eTable 8). Among term-born children, the HR of ASD for mothers with RA compared to mothers without RA was estimated at 1.18 (95% CI 0.84–1.66); among pre-term at 1.44 (95% CI 0.81–2.56) and among post-term at 2.17 (95% CI 1.36–3.44) (Table 4). There was no support for mediation of risk through preterm or post-term birth (online Supplementary eTable 9).

**Table 4.** Relative risk of ASD in offspring of mother with RA before delivery, compared to offspring of mothers without RA, by gestational age

Gestational age	ASD rate (per 100 000 person year)	ASD cases (person-years)	Number of subjects	Model 1 HR (95% CI)	Model 3 HR (95% CI)
Preterm (<37 weeks)					
Maternal RA	425.5	12 (2820)	370	1.50 (0.85–2.64)	1.44 (0.81–2.56)
No maternal RA	268.8	2446 (909 835)	87 982	Reference	Reference
Term (37–40 weeks)					
Maternal RA	217.8	39 (17 904)	2572	1.20 (0.85–1.68)	1.18 (0.84–1.66)
No maternal RA	180.8	19 121 (10 577 890)	1 035 528	Reference	Reference
Post-term (>40 weeks)					
Maternal RA	385.5	19 (4928)	687	2.08 (1.30–3.32)	2.17 (1.36–3.44)
No maternal RA	188.0	7325 (3 895 366)	380 398	Reference	Reference

Abbreviations: RA, Rheumatoid Arthritis; ASD, Autism Spectrum Disorders; HR, Hazard Ratio; 95% CI, 95% Confidence Interval.

Note: Relative risks were quantified by hazard ratios with 95% confidence intervals fitting Cox proportional hazard regression models with robust standard errors.

Model 1: Adjusted for birth year by natural cubic splines with 5 knots; Model 3: Additionally adjusted for maternal and paternal age at delivery (by natural cubic splines), maternal and paternal education at delivery (categorized; < 9 years of primary education, 9 years of primary education, 1–2 years of secondary school education, 3 years of secondary school education, 1–2 years of postgraduate education, ⩾ 3 years of postgraduate education, PhD education), maternal and paternal income at delivery (by natural cubic splines), maternal and paternal psychiatric diagnoses at delivery (yes/ no).

There was a statistically significant interaction between maternal smoking and RA. The HR was estimated at 1.55 (95% CI 1.18–2.04) among children of non-smoking mothers, and at 0.80 (95% CI 0.30–2.13) among children of smoking mothers (online Supplementary eTable 10). There was a statistically significant interaction between maternal BMI and RA. The HR was estimated at 3.28 (95% CI 1.35–8.01) for underweight mothers, and at 2.34 (95% CI 1.41–3.89) for obese, but not statistically significant for overweight and normal weight mothers (online Supplementary eTable 11).

## Discussion

In this large prospective population-based cohort study, the risk of ASD increased in offspring exposed to maternal RA before delivery, compared to offspring of mothers without RA before delivery, and more pronounced for seronegative RA. A null association was observed between maternal RA and offspring ASD when RA was diagnosed after delivery, for paternal RA, or for maternal-sister RA. The risk was more pronounced in seronegative RA, post-term births, and offspring of underweight, overweight, or non-smoking mothers.

Collectively, this indicates an increased ASD risk specific to RA exposure before delivery. Maternal arthralgia a non-inflammatory condition, displayed comparably high risks for ASD as did maternal RA, irrespective of the timing of the arthralgia diagnosis. This suggests that alternative, or additional, pathways of risk than autoimmunity/inflammation are present in RA.

Only few studies on the relationship between RA and ASD exist (online Supplementary eTable 12). Two studies (Croen et al., 2019; Tsai et al., 2018), with limited statistical power, reported a null association between maternal RA and offspring ASD risk. A large Danish cohort study (Rom et al., 2018) examined ASD risk without separating maternal RA before from after delivery (nor by RA subtype), and reported increased ASD risk in offspring to mothers with RA diagnosed at any time or after delivery. In our study, we separately examined RA diagnosed after birth as a time-varying exposure and did not observe an association with offspring ASD. In contrast to maternal RA, offspring of maternal sisters, and fathers with RA, before delivery were not at increased risk. This suggests that familial factors play a secondary role compared to maternal RA during pregnancy. This finding is supported by a previous US case–control study where, similar to our study, no association was observed for paternal history of autoimmune conditions for ASD. In contrast, the previous Danish study, where included RA diagosis after delivery, concluded that paternal RA was also associated with a higher risk of ASD in offspring. Notably, for the first time, we separately examined seropositive and seronegative RA. Although the point estimates of the ASD risk were elevated for both seropositive and seronegative RA, the association was only statistically significant for seronegative RA. However, considering the very low prevalence of RA and ASD in the population, this finding needs to be evaluated in the future studies.

Strengths of our study include the large population-based cohort design with prospective ascertainment of RA and ASD, diagnosed by clinical specialists and with extensive previous use in etiological research. Furthermore, we separated maternal RA before delivery from RA only diagnosed after delivery. Selection bias was minimized by utilizing data from a publicly financed health care system with equal access and close to complete follow-up. Our rich database allowed us to address confounding and modifying factors, and additionally, to examine the specificity of RA by including a negative control group, i.e. maternal arthralgia. As we know, our study is the first study to examine ASD risk by serostatus.

Our study also has several limitations. Despite the large sample size, statistical precision may be limited in subgroups. To address potential concerns about bias due to sparse data, we applied Firth's penalized likelihood. We used natural cubic splines to reduce residual confounding potentially introduced when categorizing measures on a continuous scale, but this may raise concern for overparameterization in the presence of sparse data. However, natural cubic splines are less sensitive to data on the tails of the distribution than, for instance, b-splines. Arthralgia is a symptom-based diagnosis code used to define joint pain in the absence of a defined underlying medical condition such as RA. Due to availability of the data, we excluded RA, Juvenile arthritis and other arthritis/arthropathies from the arthralgia group, but not those diagnosed with, e.g., psoriatic or enteropathic arthritis. However, as the prevalence of arthralgia was 5 times higher than that of RA (RA roughly equal to that of other inflammatory arthritis), the impact of misclassification of inflammatory arthritis as arthralgia should be low. Like prior research, we had no access to information on RA activity (e.g. inflammatory status), or medication during pregnancy. Medication, maternal infections and pregnancy-related factors, e.g., mode of delivery (Yip et al., 2017), may also play a role in the causal path between maternal RA and offspring ASD. Anti-inflammatory (nonsteroidal anti-inflammatory and steroids), immunosuppressive and pain medications may adversely affect pregnancy and potentially associated with ASD risk (Liew, Ritz, Virk, & Olsen, 2016).

The mechanism frequently proposed for the association between maternal RA and ASD in the offspring is through auto-antibodies and inflammatory mediators/cytokines (Estes & McAllister, 2016; Meltzer & Van de Water, 2017; Parker-Athill & Tan, 2010; Smith, Li, Garbett, Mirnics, & Patterson, 2007). Children of mothers with RA are potentially exposed to auto-antibodies and inflammatory mediators in utero which may alter fetal brain development (Estes & McAllister, 2016; Parker-Athill & Tan, 2010; Smith et al., 2007). To examine this hypothesis, we included a control group of women with arthralgia. Risk of ASD increased also in offspring of women with arthralgia, and the risk increase was of similar magnitude to the one seen in the offspring of women with RA, suggesting other pathways of risk than inflammation/autoimmunity may underlie the RA-ASD association. One possibility is that the observed association is driven by ASD risk factors shared by women with RA and women with joint pain. Alternatively, RA and arthralgia may represent distinct etiologies, both of which are independently associated with ASD risk. For maternal RA and maternal arthralgia diagnosed first after the birth of the child, arthralgia but not RA was associated with offspring ASD. Again, suggesting that ASD risk is associated with RA exposure(s) specific to pregnancy, supports the theory that ASD originates in utero (Al-Haddad et al., 2019).

Concerning potential etiological modifiable subgroups, the association between maternal RA and offspring ASD was more pronounced in children of non-optimal gestational age suggesting increased vulnerability to maternal exposure. However, there was no support for mediation of risk through preterm or post-term birth. Smoking has mostly been associated with seropositive RA (Padyukov, Silva, Stolt, Alfredsson, & Klareskog, 2004; Regueiro et al., 2020). In accordance with a greater association for seronegative RA, we observed an association between maternal RA and offspring ASD for non-smoking mothers, but not for mothers who were smokers. There was some support of increased ASD risk in offspring to women of high or low BMI. Overweight is associated with increased inflammation (Dar et al., 2018), while underweight may indicate a less favorable health condition. Both conditions may contribute to the severity of RA and modify the adverse effect of RA to ASD. However, given the limited statistical power for most subgroup analyses, these findings should be interpreted with caution and need further investigation in further studies.

## Conclusion

In Sweden, maternal RA before delivery was associated with an increased risk of offspring ASD.

Comparable associations with ASD risk for maternal arthralgia, i.e. joint pain without inflammation/autoimmunity, suggest other pathways of risk that act jointly or independently of RA.

## Supporting information

Yin et al. supplementary material Yin et al. supplementary material

## Supplementary material

The supplementary material for this article can be found at https://doi.org/10.1017/S0033291723000855
