# Supplementary material for: Maternal rheumatoid arthritis and risk of autism in the offspring
Source: Psychol Med. Author manuscript; Available in PMC 2024 Feb 16. (PMC10593909; doi:10.1017/S0033291723000855)
Supplement: Supplementary online material [file NIHMS1895905-supplement-Supplementary_online_material.pdf]

List of contents

TABLES

eTable 1 ICD codes for autism spectrum disorders (ASD), autistic disorder (AD) and rheumatoid arthritis (RA)....3

eTable 2 Risk of ASD/AD in offspring of mothers with RA at the time of delivery, compared to offspring of mothers without RA, by more stringent RA and ASD definitions....4

eTable 3 Risk of ASD in offspring of mothers with RA before delivery, compared to offspring of mothers without RA, among singletons....5

eTable 4 Risk of ASD in offspring of mothers with RA before delivery, compared to offspring of mothers without RA, among children without birth defects....6

eTable 5 Risk of ASD in offspring of mothers with RA before delivery, compared to offspring of mothers without RA, using Firth's penalized likelihood....7

eTable 6 Risk of ASD in offspring of mothers with RA and unspecific arthralgia before and after delivery as time-varying exposure, compared to offspring of mothers without RA and arthralgia....8

eTable 7 Relative risk of ASD in offspring of mother's full-sisters with arthralgia before delivery, compared to offspring of mothers without arthralgia or RA....9

eTable 8 Risk of ASD in offspring of mothers with RA before delivery, compared to offspring of mothers without RA, by offspring sex....10

eTable 9 Mediation analysis of pre- and post-term birth....11

eTable 10 Risk of ASD in offspring of mothers with RA before delivery, compared to offspring of mothers without RA, by maternal smoking at the first trimester....13

eTable 11 Risk of ASD in offspring of mothers with RA before delivery, compared to offspring of mothers without RA, by maternal BMI at first antenatal visit....14

eTable 12 Previous studies on maternal/ familial RA history and risk of ASD in offspring (to our best knowledge)....15

FIGURES

eFigure 1 The co-occurrence of being diagnosed with RA, JIA, arthralgia and other subtypes....................17

eFigure 2 Flow diagram illustrating the identification of the study cohort....................18

eFigure 3 Side-by-side bar charts of primary covariates, comparing data for individuals with ASD born to mothers with RA, without RA, and by RA serostatus....................19

eFigure 4 Adjusted Inverse Kaplan-Meier curves for mothers with and without RA before delivery, and by seronegative and seropositive RA separately....................21

eFigure 5 Scaled Schoenfeld residual plots to assess proportional hazards assumption for the RA parameter by age at follow-up....................22

eFigure 6 Incidence rate of ASD per 10,000 person-years by maternal RA and arthralgia before and after delivery....................23

eTable 1 ICD codes for Autism Spectrum Disorders (ASD), autistic disorder (AD), rheumatoid arthritis (RA) and arthralgia (negative control)

Outcome	ICD-10	ICD-9 (Swedish version)
**Autism Spectrum Disorder**		
ASD [1]	F840, F841, F843, F845, F848, F849	
AD	F840	
**RA and related diagnoses**		
RA	M05, M06	714A, 714B, 714C, 714W
Seropositive RA [2]	M05	714A, 714B, 714C
Seronegative RA	M06	714W
Juvenile arthritis	M08	714D
Other and unspecified arthropathies	M12	
Other arthritis	M13	
**Unspecific arthralgia** [3]	M25 (excluding RA related diagnoses)	

Abbreviations: ICD: International codes of diseases; RA: rheumatoid arthritis; ASD: autism spectrum disorders; AD: autistic disorder.

Note: Main or secondary diagnoses were registered in the *Swedish National Patient Register.* [1] ASD in this study was diagnosed from the age of two years to avoid possible outcome misclassification ASD diagnoses that are not reliable during the first years after birth, until maximally up to the age of 23 years (corresponding to a maximum age at end of follow-up in 2017). [2] Seropositive RA is usually denoted by the presence of rheumatoid factor, or antibodies to citrullinated peptides; Seronegative RA lacks these markers. For serotype in this study, we used the mother's all available RA diagnoses up to delivery and defined seropositive or seronegative status as the most common status. If equally frequent, we used the last diagnosis to define the RA subtype. Similar for RA after delivery. [3] Due to availability of the data, M07 (Enteropathic arthropathies) was not excluded from arthralgia group, however due to rare incidence, could not drive the nature of the whole diagnostic group.
The ICD-9 codes with suffix B/W/A/C/D are Swedish specific diagnoses.

eTable 2 Risk of ASD/AD in offspring of mothers with RA before delivery, compared to offspring of mothers without RA, by three RA definitions

Analysis group (All diagnoses before delivery)	Rate (per 100,000 person year)	ASD cases (person years)	Number of subjects	Model 1 HR (95% CI)	Model 2 HR (95% CI)	Model 3 HR (95% CI)
**ASD**						
**At least one RA diagnosis**						
Maternal RA	272.9	70 (25,652)	3,629	1.45 (1.13-1.85)	1.46 (1.14-1.87)	1.43 (1.11-1.84)
No maternal RA	187.8	28,892 (15,383,092)	1,503,908	Reference	Reference	Reference
**At least two RA diagnoses**						
Maternal RA	268.3	43 (16,026)	2,468	1.43 (1.04-1.96)	1.46 (1.06-2.01)	1.44 (1.05-1.98)
No maternal RA	187.9	28,919 (15,392,718)	1,505,069	Reference	Reference	Reference
**AD**						
**At least one RA diagnosis**						
Maternal RA	167.0	43 (25,743)	3,629	1.53 (1.11-2.11)	1.53 (1.11-2.11)	1.50 (1.09-2.07)
No maternal RA	90.2	13,947 (15,454,663)	1,503,908	Reference	Reference	Reference
**At least two RA diagnosis**						
Maternal RA	174.3	28 (16,065)	2,468	1.52 (1.02-2.26)	1.55 (1.04-2.31)	1.53 (1.03-2.27)
No maternal RA	90.3	13,962 (15,464,341)	1,5050,69	Reference	Reference	Reference

Abbreviations: RA: rheumatoid arthritis; ASD: autism spectrum disorders; AD: autistic disorder; HR: Hazard Ratio; CI: Confidence interval.

Note: Estimates were calculated by hazard ratios with 95% confidence interval using Cox regression models. **Model 1**: Adjusted for birth year by natural cubic splines with 5 knots; **Model 2**: Additionally adjusted for maternal and paternal education at delivery (categorized; < 9 years of primary education, 9 years of primary education, 1-2 years of secondary school education, 3 years of secondary school education, 1-2 years of postgraduate education, ≥ 3 years of postgraduate education, PhD education), maternal and paternal age at delivery (by natural cubic splines), maternal and paternal income at delivery (by natural cubic splines); **Model 3**: Additionally adjusted for maternal and paternal psychiatric diagnoses at delivery (yes/ no).

eTable 3 Risk of ASD in offspring of mothers with RA before delivery, compared to offspring of mothers without RA, among singletons

Maternal RA	Rate (per 100,000 person year)	ASD cases (person years)	Number of subjects	Model 1 HR (95% CI)	Model 2 HR (95% CI)	Model 3 HR (95% CI)
Yes	268.8	66 (24,553)	3473	1.43 (1.10-1.85)	1.44 (1.11-1.87)	1.42 (1.10-1.84)
No	188.2	28,068 (14,915,982)	1,459,373	Reference	Reference	Reference

Abbreviations: RA: rheumatoid arthritis; ASD: autism spectrum disorders; HR: Hazard Ratio; CI: Confidence interval.

Note: Analysis restricted to singletons in the cohort. Estimates were calculated by hazard ratios with 95% confidence interval using Cox proportional hazard regression models. **Model 1**: Adjusted for birth year by natural cubic splines with 5 knots; **Model 2**: Additionally adjusted for maternal and paternal education at delivery (categorized; < 9 years of primary education, 9 years of primary education, 1-2 years of secondary school education, 3 years of secondary school education, 1-2 years of postgraduate education, ≥ 3 years of postgraduate education, PhD education), maternal and paternal age at delivery (by natural cubic splines), maternal and paternal income at delivery (by natural cubic splines); **Model 3**: Additionally adjusted for maternal and paternal psychiatric diagnoses at delivery (yes/ no).

eTable 4 Risk of ASD in offspring of mothers with RA before delivery, compared to offspring of mothers without RA, among children without birth defects

Maternal RA	Rate (per 100,000 person year)	ASD cases (person years)	Number of subjects	Model 1 HR (95% CI)	Model 2 HR (95% CI)	Model 3 HR (95% CI)
Yes	264.2	65 (24,602)	3,486	1.43 (1.11-1.86)	1.45 (1.12-1.88)	1.42 (1.10-1.85)
No	183.9	27,286 (14,837,248)	1,450,761	Reference	Reference	Reference

Abbreviations: RA: rheumatoid arthritis; ASD: autism spectrum disorders; HR: Hazard Ratio; CI: Confidence interval.

Note: Analysis restricted to children without birth defects. Estimates were calculated by hazard ratios with 95% confidence interval using Cox proportional hazard regression models. **Model 1**: Adjusted for birth year by natural cubic splines with 5 knots; **Model 2**: Additionally adjusted for maternal and paternal education at delivery (categorized; < 9 years of primary education, 9 years of primary education, 1-2 years of secondary school education, 3 years of secondary school education, 1-2 years of postgraduate education, ≥ 3 years of postgraduate education, PhD education), maternal and paternal age at delivery (by natural cubic splines), maternal and paternal income at delivery (by natural cubic splines); **Model 3**: Additionally adjusted for maternal and paternal psychiatric diagnoses at delivery (yes/ no).

eTable 5 Risk of ASD in offspring of mothers with RA before delivery, compared to offspring of mothers without RA, using Firth's penalized likelihood

Maternal RA	Rate, per 100,000 person year	ASD cases (person years)	Number of subjects	Model 1 HR (95% CI)	Model 2 HR (95% CI)	Model 3 HR (95% CI)
Yes	272.9	70 (25,652)	3,629	1.46 (1.15-1.84)	1.47 (1.16-1.86)	1.44 (1.14-1.82)
No	187.8	28,892 (15,383,092)	1,503,908	Reference	Reference	Reference

Abbreviations: RA: rheumatoid arthritis; ASD: autism spectrum disorders; HR: Hazard Ratio; CI: Confidence interval.

Note: Estimates were calculated by hazard ratios with 95% confidence interval using Cox proportional hazard regression models. To address potential concerns about sparse data and small sample biases of the parameter estimates, Firth's penalized likelihood was applied. **Model 1**: Adjusted for birth year by natural cubic splines with 5 knots; **Model 2**: Additionally adjusted for maternal and paternal education at delivery (categorized; < 9 years of primary education, 9 years of primary education, 1-2 years of secondary school education, 3 years of secondary school education, 1-2 years of postgraduate education, ≥ 3 years of postgraduate education, PhD education), maternal and paternal age at delivery (by natural cubic splines), maternal and paternal income at delivery (by natural cubic splines); **Model 3**: Additionally adjusted for maternal and paternal psychiatric diagnoses at delivery (yes/ no).

eTable 6 Risk of ASD in offspring of mothers with RA and unspecific arthralgia before and after delivery as time-varying exposure, compared to offspring of mothers without RA and arthralgia

Analysis group	Rate, per 100,000 person year	ASD cases (person years)	Number of subjects	Model 1 IRR (95% CI)	Model 3 IRR (95% CI)
**Exposure **before** delivery**					
RA before delivery	278.5	70 (25,133)	3,617	1.50 (1.19-1.90)	1.49 (1.18-1.88)
Arthralgia before delivery	280.6	240 (85,519)	17,339	1.57 (1.38-1.79)	1.41 (1.24-1.60)
**Exposure **after** delivery**					
RA after delivery	290.4	125 (43,047)	449	1.19 (1.00-1.42)	1.13 (0.95-1.35)
Arthralgia after delivery	379.1	1,042 (274,843)	4,321	1.43 (1.35-1.52)	1.36 (1.27-1.44)
**No RA or arthralgia**	183.4	27,485 (14,967,679)	1,481,811	Reference	Reference
RA before vs after delivery	-	-	-	1.26 (0.94-1.69)	1.31 (0.98-1.76)
Arthralgia before vs after delivery	-	-	-	1.10 (0.95-1.27)	1.04 (0.90-1.20)

Abbreviations: RA: rheumatoid arthritis; ASD: autism spectrum disorders; IRR: Incident rate ratios; CI: Confidence interval.

Note: Estimates of relative risk with 95% confidence interval was calculated using Poisson regression models, including the diagnosis of RA and unspecific arthralgia as a time-varying exposure. **Model 1**: Adjusted for birth year by natural cubic splines with 5 knots; **Model 3**: Additionally adjusted for maternal and paternal education at delivery (categorized; < 9 years of primary education, 9 years of primary education, 1-2 years of secondary school education, 3 years of secondary school education, 1-2 years of postgraduate education, ≥ 3 years of postgraduate education, PhD education), maternal and paternal age at delivery (by natural cubic splines), maternal and paternal income at delivery (by natural cubic splines), maternal and paternal psychiatric diagnoses at delivery (yes/ no).

eTable 7 Relative risk of ASD in offspring of mother's full-sisters with arthralgia before delivery, compared to offspring of mothers without arthralgia or RA.

Analysis group	Rate, per 100,000 person year	ASD cases (person years)	Number of subjects	Model 1 HR (95% CI)	Model 3 HR (95% CI)
Sisters to maternal arthralgia *	206.9	148 (71,537)	8,299	1.09 (0.92-1.29)	1.06 (0.89-1.25)
No maternal RA or arthralgia	187.3	28,505 (15,221,571)	1,480,745	Reference	Reference

Abbreviations: RA: rheumatoid arthritis; ASD: autism spectrum disorders; HR Hazard Ratio; CI 95% Confidence Interval.

Note: Relative risks were quantified by hazard ratios with their associated 95% confidence interval fitting Cox proportional hazard regression models with robust standard errors. *Children of full sisters to mothers with arthralgia before delivery versus children of RA/arthragia-free mothers. **Model 1**: Adjusted for birth year by natural cubic splines with 5 knots; **Model 3**: Additionally adjusted for maternal and paternal education at delivery (categorized; < 9 years of primary education, 9 years of primary education, 1-2 years of secondary school education, 3 years of secondary school education, 1-2 years of postgraduate education, ≥ 3 years of postgraduate education, PhD education), maternal and paternal age at delivery (by natural cubic splines), maternal and paternal income at delivery (by natural cubic splines), maternal and paternal psychiatric diagnoses at delivery (yes/ no).

eTable 8 Relative risk of ASD in offspring of mother with RA before delivery, compared to offspring of mothers without RA, by offspring sex

Analysis group	Rate, per 100,000 person year	ASD cases (person years)	Number of Subjects	Model 1 HR (95% CI)	Model 2 HR (95% CI)	Model 3 HR (95% CI)
**At least one RA diagnosis**						
**Subgroup: Males**						
Maternal RA	411.8	52 (12,627)	1,813	1.53 (1.16-2.03)	1.56 (1.18-2.06)	1.54 (1.16-2.03)
No maternal RA	255.8	20,170 (7,885,909)	773,899	Reference	Reference	Reference
**Subgroup: Females**						
Maternal RA	138.2	18 (13,025)	1,816	1.33 (0.84-2.11)	1.33 (0.84-2.12)	1.29 (0.81-2.06)
No maternal RA	116.3	8,722 (7,497,183)	730,009	Reference	Reference	Reference
**At least two RA diagnosis**						
**Subgroup: Males**						
Maternal RA	371.9	29 (7,798)	1,226	1.36 (0.94-1.99)	1.40 (0.96-2.04)	1.40 (0.96-2.03)
No maternal RA	255.9	20,193 (7,890,738)	774,486	Reference	Reference	Reference
**Subgroup: Females**						
Maternal RA	170.1	14 (8,228)	1242	1.71 (1.01-2.88)	1.76 (1.04-2.97)	1.69 (1.00-2.86)
No maternal RA	116.3	8,726 (7,501,980)	730,583	Reference	Reference	Reference

Abbreviations: RA: rheumatoid arthritis; ASD: autism spectrum disorders; HR Hazard Ratio; CI 95% Confidence Interval.

Note: Estimates were calculated by hazard ratios with 95% confidence interval using Cox proportional hazard regression models. **Model 1**: Adjusted for birth year by natural cubic splines with 5 knots; **Model 2**: Additionally adjusted for maternal and paternal education at delivery (categorized; < 9 years of primary education, 9 years of primary education, 1-2 years of secondary school education, 3 years of secondary school education, 1-2 years of postgraduate education, ≥ 3 years of postgraduate education, PhD education), maternal and paternal age at delivery (by natural cubic splines), maternal and paternal income at delivery (by natural cubic splines); **Model 3**: Additionally adjusted for maternal and paternal psychiatric diagnoses at delivery (yes/ no). P value for interaction term between offspring sex and maternal RA: P=0.22.

eTable 9 Mediation analysis of pre- and post-term birth

**Mediation analysis - Effect measures**	**Odds-Ratios (95% Confidence intervals)**			
	**Preterm**		**Post-term**	
	**Crude**	**Adjusted**	**Crude**	**Adjusted**
Total Effect	1.35 (0.99-1.76)	1.21 (0.85-1.61)	1.37 (1.00-1.81)	1.37 (1.01-1.81)
Controlled Direct Effect (CDE)	1.20 (0.83-1.62)	1.17 (0.82-1.59)	1.21 (0.83-1.69)	1.18 (0.81-1.64)
Natural Direct Effect (NDE)	1.28 (0.93-1.68)	1.20 (0.85-1.61)	1.41 (1.04-1.86)	1.41 (1.03-1.86)
Natural Indirect Effect (NIE)	1.06 (1.00-1.14)*	1.00 (1.00-1.02)	0.97 (0.93-1.01)	0.97 (0.94-1.00)

**P* value=0.0665.

METHODS

Mediation analysis of preterm vs term born, and post-term vs term birth were performed by approximating our Cox models with logistic regression, and fitting Natural Effects Models [1, 2]. We used the SAS software Proc Causalmed SAS/Stat 15.2. These analyses are using two logistic regression models, one outcome-model including RA as a predictor for offspring ASD risk and one mediation-model including RA as a predictor for preterm birth (or post-term).

From the logistic regression models we calculated three different odds ratios describing the mediating role of preterm birth. In short, the controlled direct effect (**CDE**) for a subject is defined as the difference between the counterfactual outcomes at the two treatment levels when an intervention sets the mediator to a particular level M = m. That is, here, outcome difference for preterm children born to RA vs RA free mothers. The natural direct effect (**NDE**) for a subject is defined as the difference between the counterfactual outcomes at the two treatment levels when an intervention sets the mediator value to M = M0, which is the natural level of the mediator when there is no treatment. That is, here, outcome difference for term born children, comparing offspring born to RA vs RA free mothers. The natural indirect effect (**NIE**) for a subject is defined as the difference between the counterfactual outcomes at the two mediator levels at M1 and M0 when an intervention sets the treatment to T = 1. That is, here, outcome difference for preterm minus term born among children with RA mother at birth. The controlled level m of the mediator variable does not change the estimates of the natural direct effect (NDE), or the natural indirect effect (NIE), but it does change the estimates of

the controlled direct effect (CDE). In the crude model, we adjusted for birth year (categories 1995-1999 2000-2004 2005-2009 2010-2015). In the full-adjusted model, we additionally adjusted confounding by maternal and paternal age (years), maternal BMI (<18.5, 18.5-24.9, 25-29.9 and >=30), Size for gestational age ("Small", "Appropriate", "Large"), Maternal and paternal education (<9 yrs, 9 yrs, 2nd level 1-2 yrs, 2nd level 3 yrs, PhD, Post 2nd level <3 yrs, Post 2nd level =>3 yrs), Maternal and paternal income (SEK/year) and Maternal and psychiatric history (yes/no), all defined at delivery. We calculated percentile based 95% bootstrap confidence intervals [3].

RESULTS

Assessed through the estimated natural indirect effects, there was no support for a mediating effect by preterm birth, OR=1.00 (95% CI: 1.00-1.02), or by post-term birth, OR=0.97 (95% CI: 0.94-1.00).

REFERENCES

1 VanderWeele TJ. A unification of mediation and interaction: a four-way decomposition. Epidemiology. 2014;25(5):749-761. doi:10.1097/EDE.0000000000000121
2 VanderWeele T. Explanation in Causal Inference: Methods for Mediation and Interaction. Oxford University Press; 2015.
3 Efron B, Tibshirani RJ. An Introduction to the Bootstrap. 1st ed. Chapman and Hall/CRC; 1994.

eTable 10 Risk of ASD in offspring of mothers with RA before delivery, compared to offspring of mothers without RA, by subgroups of maternal smoking during pregnancy

Analysis subgroup	Rate (per 100,000 person year)	ASD cases (person years)	Number of subjects	Model 1 HR (95% CI)	Model 3 HR (95% CI)
No maternal smoking					
Maternal RA	271.7	57 (20,982)	3117	1.55 (1.18-2.03)	1.55 (1.18-2.04)
No maternal RA	172.7	20,934 (12,120,670)	1240332	Reference	Reference
Maternal smoking					
Maternal RA	284.7	4 (1,405)	155	0.88 (0.34-2.32)	0.80 (0.30-2.13)
No maternal RA	305.6	3,658 (1,196,951)	94256	Reference	Reference

Abbreviations: RA: rheumatoid arthritis; ASD: autism spectrum disorders. HR Hazard Ratio; CI 95% Confidence Interval.

Note: Analysis restricted to individuals without missing value in maternal smoking during pregnancy. Estimates were calculated by hazard ratios with 95% confidence interval using Cox proportional hazard regression models. An interaction term between maternal smoking and maternal RA was included (P <.0001) and only the strata-specific effect is shown. **Model 1**: Adjusted for birth year by natural cubic splines with 5 knots; **Model 3**: Additionally adjusted for maternal and paternal education at delivery (categorized; < 9 years of primary education, 9 years of primary education, 1-2 years of secondary school education, 3 years of secondary school education, 1-2 years of postgraduate education, ≥ 3 years of postgraduate education, PhD education), maternal and paternal age at delivery (by natural cubic splines), maternal and paternal income at delivery (by natural cubic splines), maternal and paternal psychiatric diagnoses at delivery (yes/ no).

eTable 11 Risk of ASD in offspring of mothers with RA before delivery, compared to offspring of mothers without RA, in subgroups of maternal BMI at first antenatal visit

Categories of maternal BMI	Rate, per 100,000 person year	Cases (person-years)	Number of Subjects	Model 1 HR (95% CI)	Model 3 HR (95% CI)
Underweight (BMI<18.5 kg/m2)					
Maternal RA	591.9	5 (845)	97	2.77 (1.14-6.76)	3.28 (1.35-8.01)
No maternal RA	215.8	658 (304,968)	30,001	Reference	Reference
Normal weight (BMI 18.5-24.9 kg/m2)					
Maternal RA	213.0	30 (14,087)	2012	1.34 (0.94-1.91)	1.33 (0.93-1.90)
No maternal RA	158.5	13,703 (8,645,981)	849,365	Reference	Reference
Overweight (BMI 25-29.9 kg/m2)					
Maternal RA	224.9	12 (5,336)	797	1.12 (0.64-1.97)	1.09 (0.62-1.92)
No maternal RA	202.3	6,433 (3,180,052)	321,233	Reference	Reference
Obesity (BMI≥30 kg/m2)					
Maternal RA	671.6	16 (2,382)	391	2.27 (1.37-3.77)	2.34 (1.41-3.89)
No maternal RA	303.0	4,069 (1,343,075)	146,108	Reference	Reference

Abbreviations: RA: rheumatoid arthritis; ASD: autism spectrum disorders. HR Hazard Ratio; CI 95% Confidence Interval; BMI: Body Mass Index

Note: Analysis restricted to individuals without missing value in maternal BMI at first antenatal visit. Estimates were calculated by hazard ratios with 95% confidence interval using Cox proportional hazard regression models. An interaction term between maternal BMI category and maternal RA was included (P <0.0001) and only the strata-specific effect is shown. **Model 1**: Adjusted for birth year by natural cubic splines with 5 knots; **Model 3**: Additionally adjusted for maternal and paternal education at delivery (categorized; < 9 years of primary education, 9 years of primary education, 1-2 years of secondary school education, 3 years of secondary school education, 1-2 years of postgraduate education, ≥ 3 years of postgraduate education, PhD education), maternal and paternal age at delivery (by natural cubic splines), maternal and paternal income at delivery (by natural cubic splines), maternal and paternal psychiatric diagnoses at delivery (yes/ no).

eTable 12 Previous studies on maternal/ familial RA history and risk of ASD in offspring (to our best knowledge) ordered chronologically

**Study**	**Country Year**	**Design**	**Partici-pants**	**RA diagnosis Timing**	**Findings**	**Covariates**
Croen et al.	US 2019	Case-control	Case 663 control 915	Self-reported or in medical records<br><br>**Timing**: Before delivery	Any maternal autoimmune during pregnancy: OR=1.29 (CI: 0.97-1.70). Paternal history and any familial history (maternal, paternal and siblings any time): null.<br><br>Low prevalence of RA precluding further analyses.	Maternal age and education at date of delivery, maternal race-ethnicity, household income at time of caregiver interview, and child sex.<br>Stratified by males and females<br>Subgroup by ASD+ID; ASD+ regression; ASD severity; treatment status during pregnancy
Rom et al.	Denmark 2018	Cohort	1,917,723	NPR- Sensitivity analysis: Inpatient RA; ≥two RA<br><br>**Timing**: Before and after delivery (No risk estimates for exposure to prenatal RA due to rare cases)	RA before and after delivery: Maternal RA HR=1.31 (CI: 1.06-1.63); paternal RA 1.33 (CI: 0.97-1.82).<br><br>RA after delivery: Maternal RA HR=1.39 (CI: 1.11-1.75); paternal RA 1.45 (CI: 1.04-2.03).	Birth year, parental age, maternal education, parity, RA in the other parent.<br>Supplementary analysis: Adjusted for offspring sex, maternal smoking, gestational age, birth weight, Apgar score at 5 minutes and epilepsy. Restricted to the period 1994-2010 and to 1977-2007 (Danish Psychiatric Central Registry); inpatient RA and ≥two RA; firstborn; for children without cerebral palsy; for children without epilepsy.

Abbreviations: NPR: Diagnosis was obtained from National Patient Register; RA: rheumatoid arthritis; ASD: autism spectrum disorders; HR: Hazard ratio; OR: Odds ratio; CI: 95% confidence interval.

eTable 12 (Continued)

Study	Country Year	Design	Partici-pants	RA diagnosis Timing	Findings	Covariates
Tsai et al.	Taiwan 2018	Cohort	1,893,244	NPR<br><br>**Timing**: Before delivery	Maternal RA HR=1.42 (CI: 0.60–3.40) (Very few cases: 5 ASD of 673 born to RA mothers)	Maternal age, 1 & 5-minute Apgar score, mode of delivery, offspring sex, gestational age, birth weight and place of residence.
Atladóttir et al.	Denmark<br><br>2009	Cohort	689,196	NPR<br><br>**Timing**: Unspecified (RA as time-varying exposure but not separate before or after delivery)	Maternal RA RR=1.70 (CI: 1.07–2.54);<br><br>Parental or sibling RA RR=1.32 (CI: 0.88–1.90)	Calendar year, age at diagnosis, interaction of gender, place of birth, parental age at birth. A stratified analysis restricted to children with a gestational age of ≥37weeks, a birthweight of ≥2500g, and a 5-minute Apgar score of ≥6.
Comi et al.	US<br><br>1999	Case control	Case 61, control 46	Questionnaire<br><br>**Timing**: Unspecified	Familial RA or/and JIA OR=2.4 (1.1–5.5); familial RA OR=2.1 (CI: 0.9–4.8) (mother, father or other family members)	Matched by offspring age and sex

Abbreviations: NPR: Diagnosis was obtained from National Patient Register; RA: rheumatoid arthritis; ASD: autism spectrum disorders; HR: Hazard ratio; OR: Odds ratio; CI: 95% confidence interval.

eFigure 1 The co-occurrence of being diagnosed with RA (green), JIA (purple), arthralgia (orange) and other subtypes (M13 blue; M12 yellow)

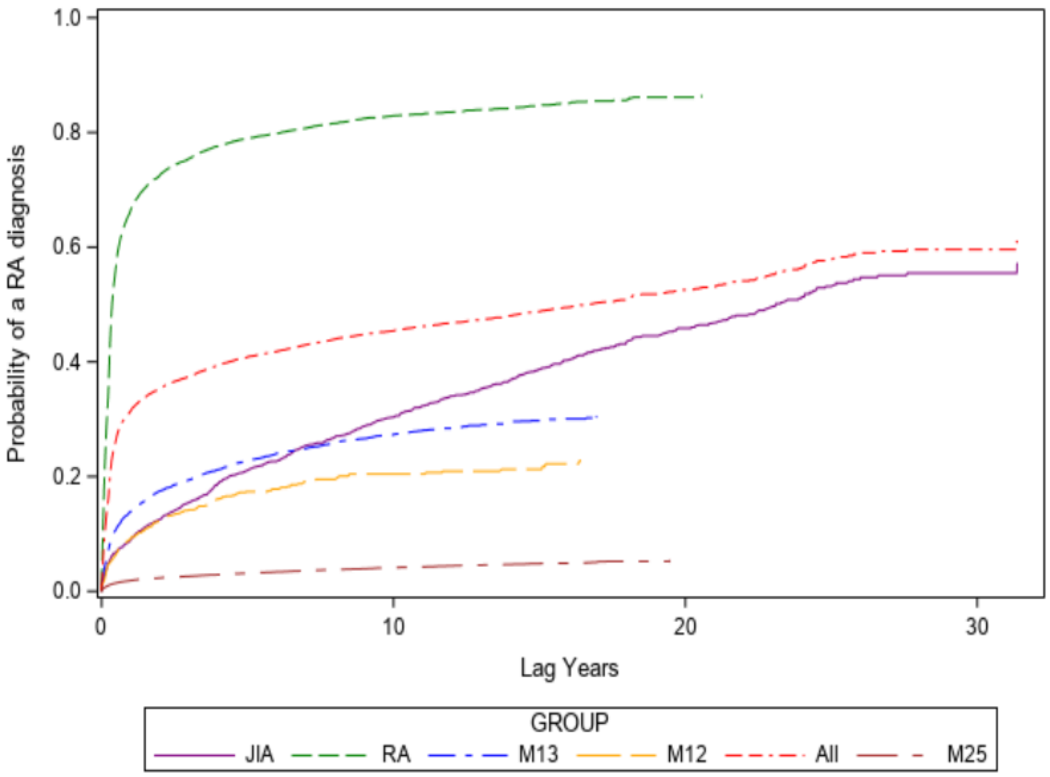

Abbreviations: RA: rheumatoid arthritis; JIA: Juvenile arthritis; ICD: International codes of diseases; M25: Unspecific arthralgia (ICD 10 code=M25, excluding ICD 10 codes of M12, M13, M05, M06, M08 and M09, as well as ICD 9 codes of 714A, 714B, 714C and 714W; M12: Other specific arthropathies (ICD 10 code=M12); M 13: Other type of arthritis (ICD 10 code=M13).

Note: The occurrence of a RA diagnosis after an initial diagnosis of RA, JIA, arthralgia and other subtypes.

eFigure 2 Flow diagram illustrating the identification of the study cohort

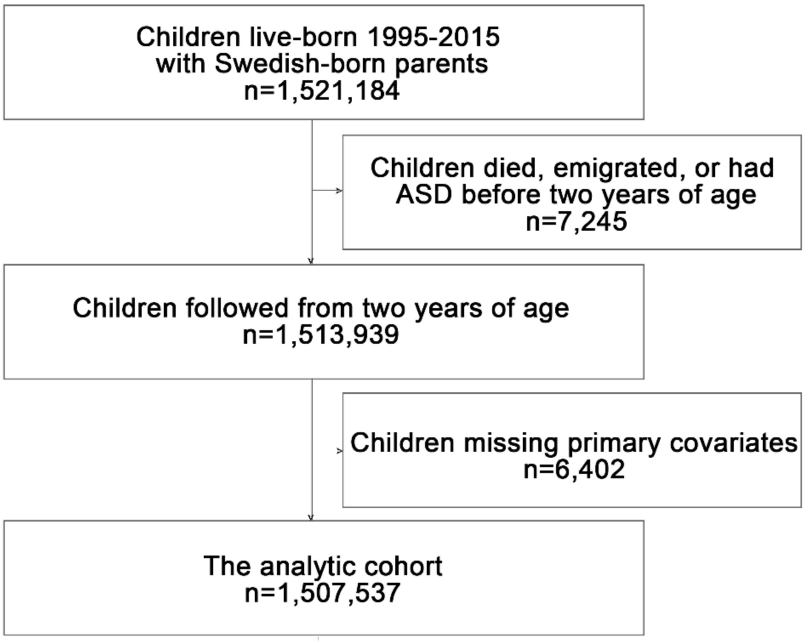

Abbreviations. n: Number of participants; ASD: Autism Spectrum Disorder

Note: Due to data protection, we only had access to date of birth by year and month, and assigned all birth dates as the first date of each month.

eFigure 3 Side-by-side bar charts of primary covariates, comparing data for individuals with ASD born to mothers with RA, without RA, and by RA serostatus

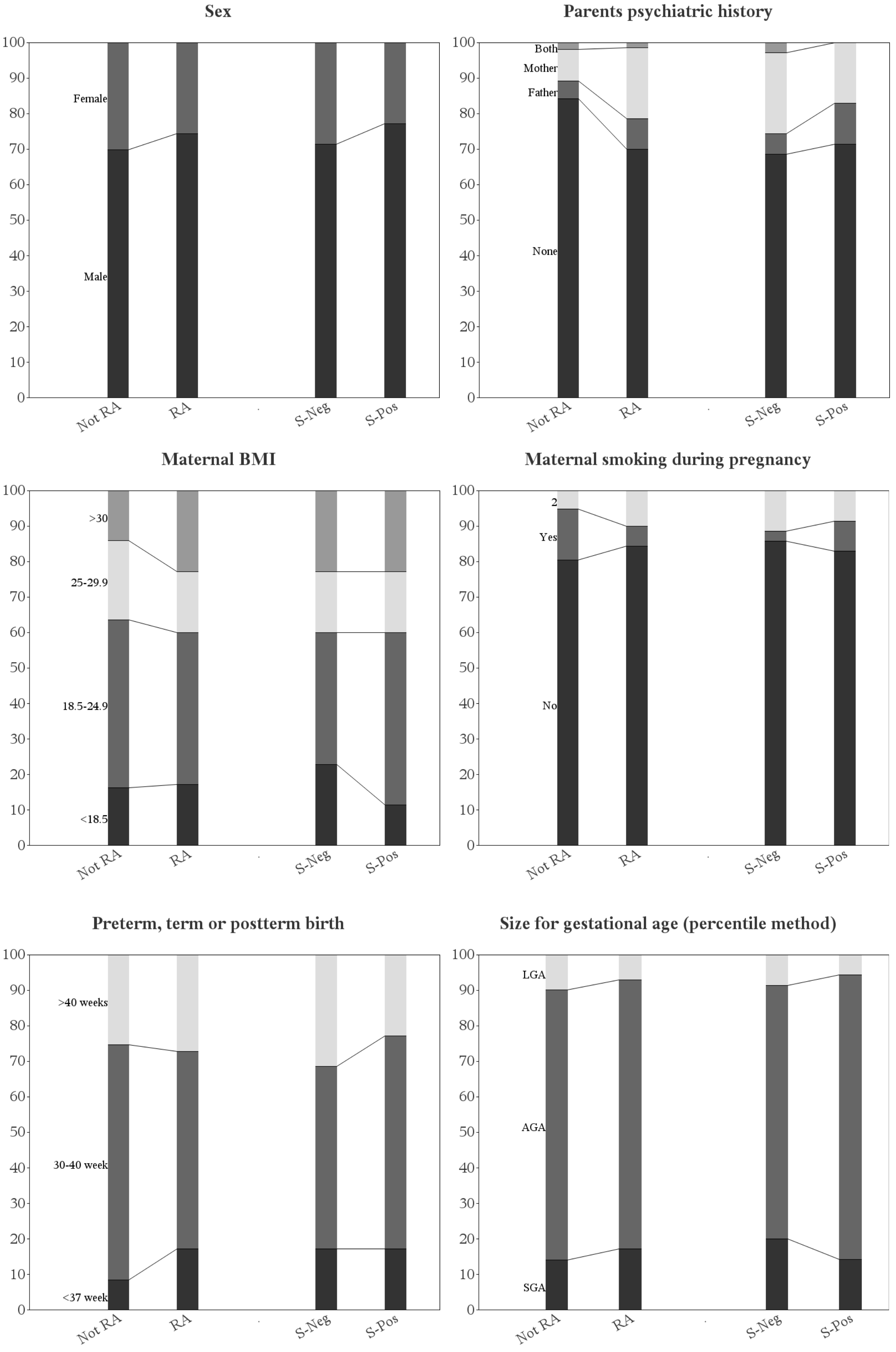

eFigure 3 (Continued)

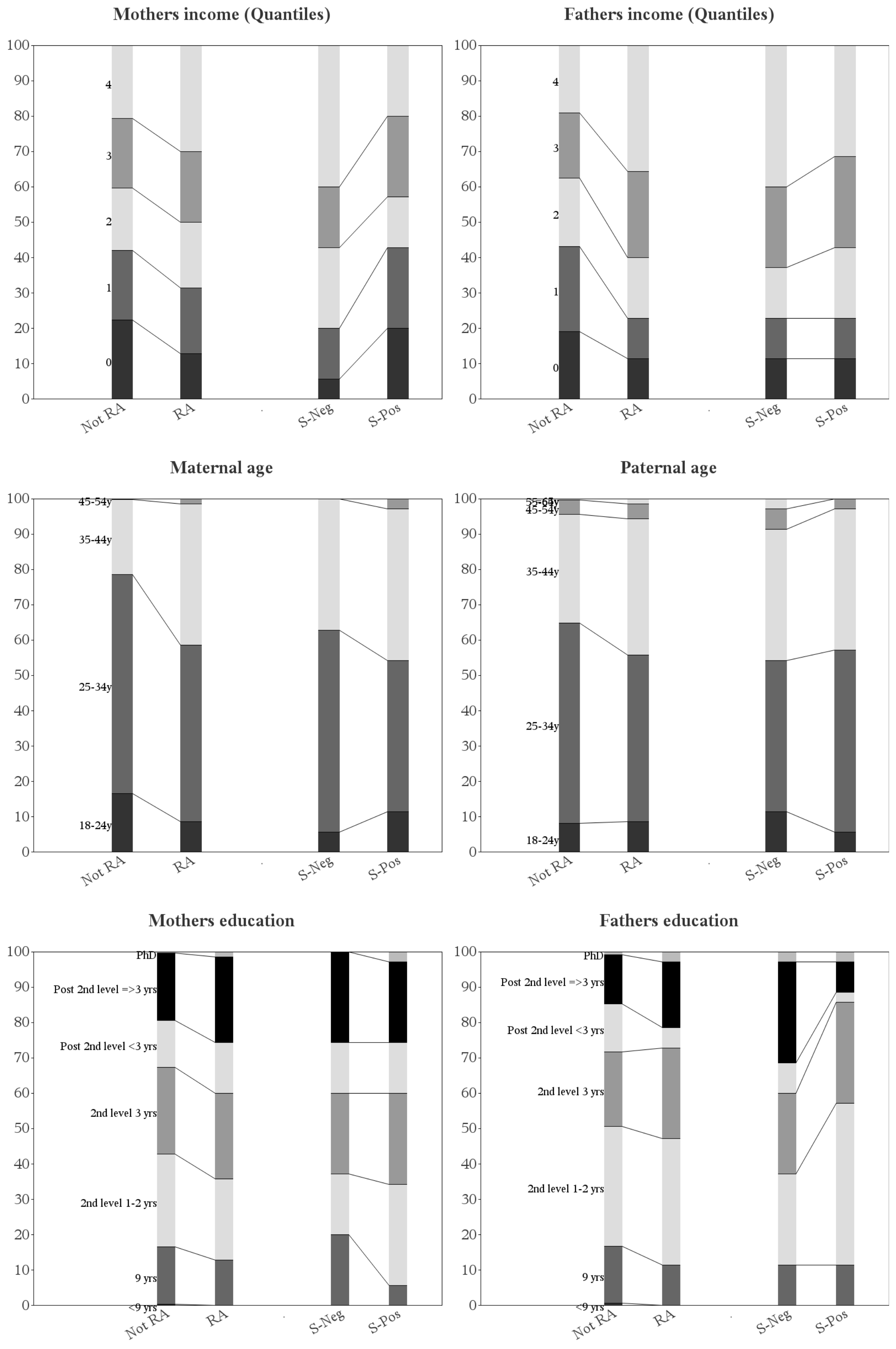

eFigure 4 Adjusted Inverse Kaplan-Meier curves for mothers with and without RA before delivery, and by seronegative and seropositive RA separately

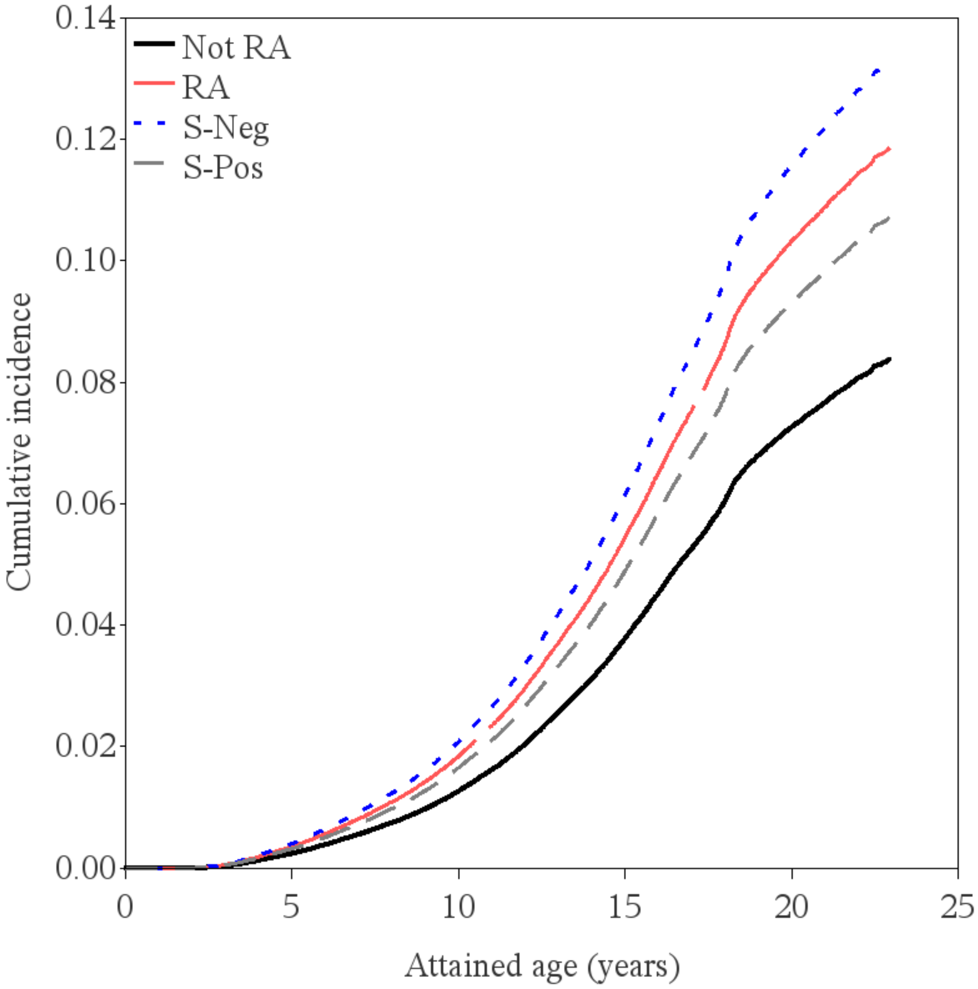

Abbreviations. RA: Rheumatoid Arthritis; S-Pos: seropositive RA; S-Neg: seronegative RA; ASD: Autism Spectrum Disorder.

Note: Inverse Kaplan-Meier curves for the cumulative incidence of ASD in the offspring, comparing mothers with any RA, seropositive RA, seronegative RA and without RA, adjusted for birth year (5-year intervals), maternal and paternal age (<20 years, 20-29 years, 30-39 years and ≥40 years), education (completed school years: 0-9 years, 10-12 years and ≥13 years), and psychiatric diagnoses (yes/no), all defined at delivery. Statistical analysis was performed using the DIRADJ option in the BASELINE statement in the SAS PROC PHREG procedure, which computes the direct adjusted survival curve that averages the estimated survival curves for covariates.

eFigure 5 Scaled Schoenfeld residual plots to assess proportional hazards assumption for the RA parameter by age at follow-up

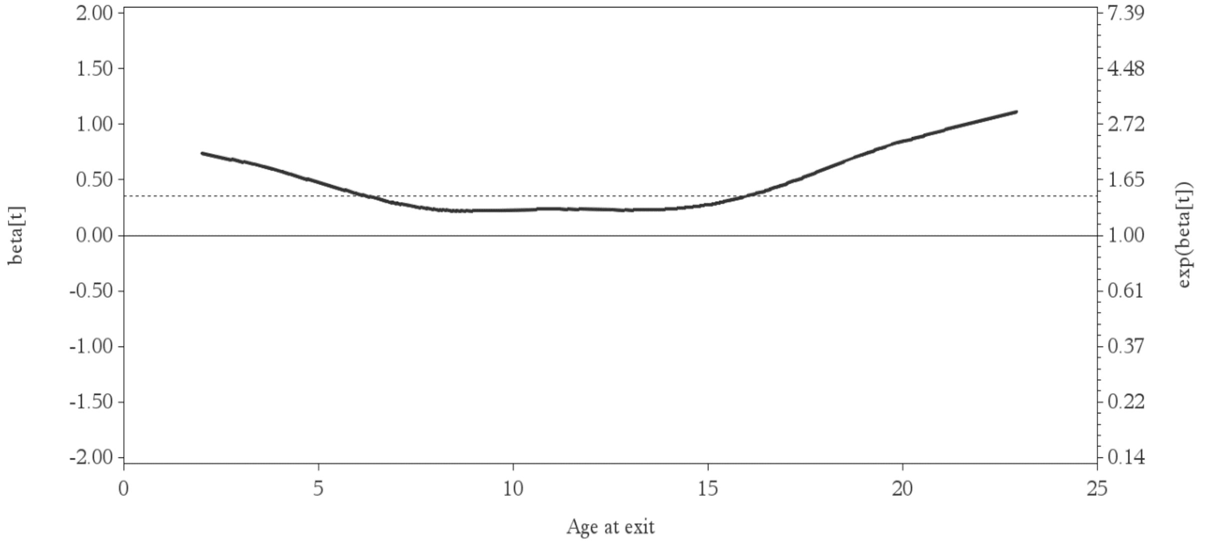

Note: The left vertical axis is on log scale and the right vertical axis is on anti-log-scale. The model adjusted for birth year by natural cubic splines with 5 knots, maternal and paternal education at delivery (categorized; < 9 years of primary education, 9 years of primary education, 1-2 years of secondary school education, 3 years of secondary school education, 1-2 years of postgraduate education, ≥ 3 years of postgraduate education, PhD education; < 9 years of primary education, 9 years of primary education, 1-2 years of secondary school education, 3 years of secondary school education, 1-2 years of postgraduate education, ≥ 3 years of postgraduate education, PhD education), maternal and paternal age at delivery (by natural cubic splines), maternal and paternal income at delivery (by natural cubic splines), maternal and paternal psychiatric diagnoses at delivery (yes/ no).

eFigure 6 Incidence rate of ASD per 10,000 person-years by maternal RA and arthralgia before and after delivery

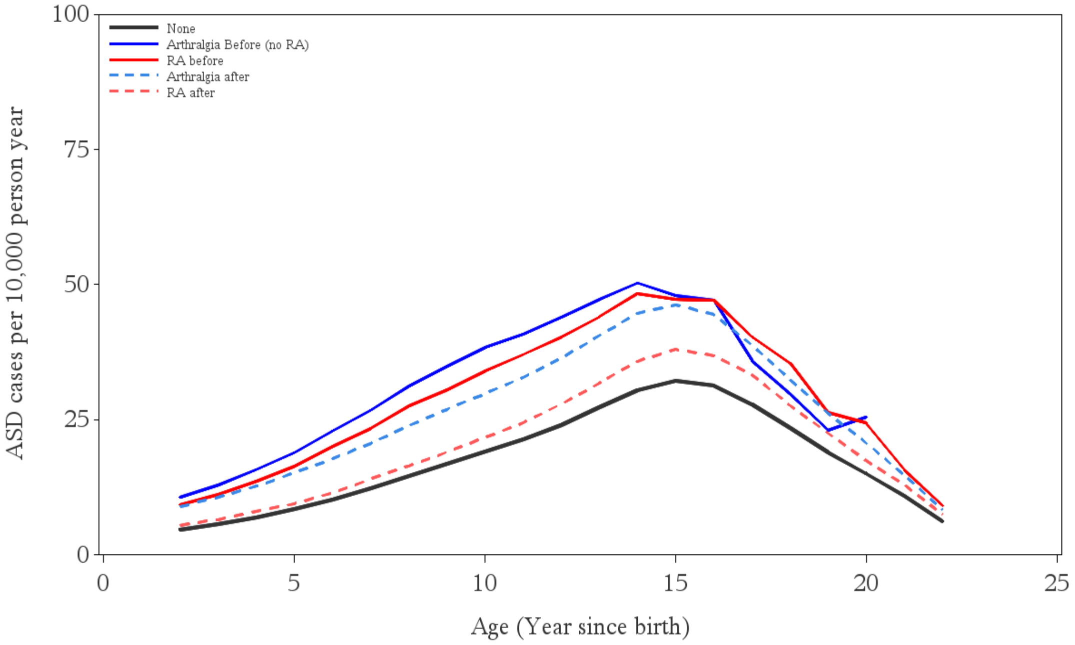

Note: Incidence rate of ASD per 10,000 person-years was predicted from poison regression model, adjusted for birth year by natural cubic splines with 5 knots, maternal and paternal education at delivery (categorized; < 9 years of primary education, 9 years of primary education, 1-2 years of secondary school education, 3 years of secondary school education, 1-2 years of postgraduate education, ≥ 3 years of postgraduate education, PhD education), maternal and paternal age at delivery (by natural cubic splines), maternal and paternal income at delivery (by natural cubic splines), maternal and paternal psychiatric diagnoses at delivery (yes/ no).
